# Supplementary material for: Neural Decoding of the Speech Envelope: Effects of Intelligibility and Spectral Degradation
Source: Trends Hear. 2024 Aug 25;28:23312165241266316. doi: 10.1177/23312165241266316 (PMC11345737; doi:10.1177/23312165241266316)
Supplement: sj-docx-1-tia-10.1177_23312165241266316 - Supplemental material for Neural Decoding of the Speech Envelope: Effects of Intelligibility and Spectral Degradation [file sj-docx-1-tia-10.1177_23312165241266316.docx]

**Appendix**

**Reaction Times**

**Table A1**

*Model selection for the dependent variable of Median Reaction Times (s). Missing values of AIC indicate the model did not converge.*

| Model | df | AIC | Formula | |
| --- | --- | --- | --- | --- |
|  |  |  |  |  |
| 1 | 3 | -9.89 | 1 | + (1 \| Participant) |
| 2 | 5 | -45 | 1 | + Spectral Degradation + (1 \| Participant) |
| 3 | 4 | -46.05 | 1 | + Language + (1 \| Participant) |
| 4 | 4 | -60.82 | 1 | + Ability to Follow + (1 \| Participant) |
| 5 | 4 | -57.57 | 1 | + Engagement + (1 \| Participant) |
| 6 | 6 | -95.71 | 1 | + Spectral Degradation + Language + (1 \| Participant) |
| 7 | 6 | -91.46 | 1 | + Spectral Degradation + Ability to Follow + (1 \| Participant) |
| 8 | 8 | -83.51 | 1 | + Spectral Degradation * Language + (1 \| Participant) |
| 9 | 11 | — |  | 1 + Spectral Degradation + Language + (1 + Spectral Degradation \| Participant) |
| 10 | 8 | -99.83 |  | 1 + Spectral Degradation + Language + (1 + Language \| Participant) |
| 11 | 14 | — |  | 1 + Spectral Degradation + Language + (1 + Spectral Degradation \| Participant) +  (Language \| Participant) |
|  |  |  |  |  |

**Table A2**

*Model formula: Median RT* ∼ *1 + Spectral Degradation + Language + (1 + Language | Participant). Standardized parameters were obtained by fitting the model on a standardized version of the dataset. 95% Confidence Intervals (CIs) and p-values were computed using a Wald t-distribution with Kenward-Roger approximation.*

| Parameter | Estimate | 95% CI | *t* | df | *p* | Std. Estimate | Std. Estimate 95% CI |  |
| --- | --- | --- | --- | --- | --- | --- | --- | --- |
|  |  |  |  |  |  |  |  |  |
| Intercept | 1.02 | [0.94,1.10] | 25.33 | 45.28 | *<* 0*.*001 | -0.56 | [-0.84,-0.28] |  |
| Vocoded | 0.05 | [0.01,0.10] | 2.43 | 150.00 | 0.016 | 0.18 | [ 0.03,0.33] |  |
| Vocoded + Blurring | 0.20 | [0.16,0.24] | 9.21 | 150.00 | *<* 0*.*001 | 0.69 | [ 0.54,0.83] |  |
| Dutch | 0.16 | [0.11,0.21] | 6.50 | 37.00 | *<* 0*.*001 | 0.55 | [ 0.38,0.72] |  |
|  |  |  |  |  |  |  |  |  |
| **Random Effects** |  |  |  |  |  |  |  |  |
|  |  |  |  |  |  |  |  |  |
| Within-Group Variance (SD) |  | 0.02 (0.13) |  |  |  |  |  |  |
| Between-Group Variance (SD) |  |  |  |  |  |  |  |  |
|  | Intercept (Participant) | 0.05 (0.22) |  |  |  |  |  |  |
|  | Slope (Language) | 0.01 (0.11) |  |  |  |  |  |  |
| Correlations |  |  |  |  |  |  |  |  |
| Groups | Participant × Language | 0.01 |  |  |  |  |  |  |
|  |  |  |  |  |  |  |  |  |
|  | Participant | 38 |  |  |  |  |  |  |
| Observations |  | 228 |  |  |  |  |  |  |
|  |  |  |  |  |  |  |  |  |
| **Model Metrics** |  |  |  |  |  |  |  |  |
|  |  |  |  |  |  |  |  |  |
| AIC | BIC | R^2^ (Conditional) | R^2^ (Marginal) | ICC | RMSE |  |  |  |
| -99.83 | -72.40 | 0.79 | 0.16 | 0.75 | 0.12 |  |  |  |
|  |  |  |  |  |  |  |  |  |

**Table A3**

*Contrast of Median Reaction Times (s) estimated marginal means for Spectral*

*Degradation. P-values are adjusted using the Bonferroni method.*

| Contrast: Spectral Degradation | | Estimate | 95% CI | SE | df | *t* | *p* |
| --- | --- | --- | --- | --- | --- | --- | --- |
|  |  |  |  |  |  |  |  |
| Unprocessed | Vocoded + Blurring | -0.20 | [-0.25,-0.15] | 0.02 | 150.00 | -9.21 | *<* 0*.*001 |
| Unprocessed | Vocoded | -0.05 | [-0.11,0.00] | 0.02 | 150.00 | -2.43 | 0.049 |
| Vocoded | Vocoded + Blurring | -0.15 | [-0.20,-0.09] | 0.02 | 150.00 | -6.78 | *<* 0*.*001 |
|  |  |  |  |  |  |  |  |

**Subject-Specific Decoding**

**Table A4**

*Model selection for the dependent variable of subject-specific Decoding Accuracy (r).*

*Missing values of AIC indicate the model did not converge.*

| Model | df | AIC | Formula | |
| --- | --- | --- | --- | --- |
|  |  |  |  |  |
| 1 | 3 | -3898.05 | 1 | + (1 \| Participant) |
| 2 | 5 | -3885.63 | 1 | + Trained: Spectral Degradation + (1 \| Participant) |
| 3 | 5 | -3913.99 | 1 | + Test: Spectral Degradation + (1 \| Participant) |
| 4 | 7 | -3901.82 | 1 | + Trained: Spectral Degradation + Test: Spectral Degradation + (1 \| Participant) |
| 5 | 11 | -3962.91 | 1 | + Trained: Spectral Degradation × Test: Spectral Degradation + (1 \| Participant) |
| 6 | 16 | — | 1 | + Trained: Spectral Degradation × Test: Spectral Degradation + (Trained: Spectral |
|  |  |  | Degradation \| Participant) | |
| 7 | 16 | -4326.58 | 1 | + Trained: Spectral Degradation × Test: Spectral Degradation + (Test: Spectral |
|  |  |  | Degradation \| Participant) | |
| 8 | 17 | -4317.53 | 1 | + Trained: Spectral Degradation × Test: Spectral Degradation + Trained: Language + |
|  |  |  | (Test: Spectral Degradation \| Participant) | |
| 9 | 17 | -4367.24 | 1 | + Trained: Spectral Degradation × Test: Spectral Degradation + Test: Language + |
|  |  |  | (Test: Spectral Degradation \| Participant) | |
| 10 | 18 | -4358.28 | 1 | + Trained: Spectral Degradation × Test: Spectral Degradation + Trained: Language + |
|  |  |  | Test: Language + (Test: Spectral Degradation \| Participant) | |
| 11 | 19 | -4373.39 | 1 | + Trained: Spectral Degradation × Test: Spectral Degradation + Trained: Language × |
|  |  |  | Test: Language + (Test: Spectral Degradation \| Participant) | |
| 12 | 22 | — | 1 | + Trained: Spectral Degradation × Test: Spectral Degradation + Trained: Language × |
|  |  |  | Test: Language + (Test: Spectral Degradation \| Participant) + (Trained: Language \| | |
|  |  |  | Participant) | |
| 13 | 22 | — | 1 | + Trained: Spectral Degradation × Test: Spectral Degradation + Trained: Language × |
|  |  |  | Test: Language + (Test: Spectral Degradation \| Participant) + (Test: Language \| | |
|  |  |  | Participant) | |
| 14 | 27 | -4310.04 | 1 | + Trained: Spectral Degradation × Test: Spectral Degradation × Trained: Language + |
|  |  |  | Trained: Language × Test: Language + (Test: Spectral Degradation \| Participant) | |
| 15 | 27 | -4319.79 | 1 | + Trained: Spectral Degradation × Test: Spectral Degradation × Test: Language + |
|  |  |  | Trained: Language × Test: Language + (Test: Spectral Degradation \| Participant) | |
| 16 | 43 | -4192.12 | 1 | + Trained: Spectral Degradation × Test: Spectral Degradation × Trained: Language × |
|  |  |  | Test: Language + (Test: Spectral Degradation \| Participant) | |
|  |  |  |  |  |

**Table A5**

*Model formula: Decoding Accuracy R Value* ∼ *1 + Trained: Spectral Degradation* × *Test: Spectral Degradation + Trained: Language* × *Test: Language + (Test: Spectral Degradation | Participant). Standardized parameters were obtained by fitting the model on a standardized version of the dataset. 95% Confidence Intervals (CIs) and p-values were computed using a Wald t-distribution with Kenward-Roger approximation.*

| Parameter | Estimate | 95% CI | *t* | df | *p* | Std. Estimate | Std. Estimate 95% CI |  |
| --- | --- | --- | --- | --- | --- | --- | --- | --- |
|  |  |  |  |  |  |  |  |  |
| (Intercept) | 0.17 | [ 0.15,0.19] | 15.84 | 45.93 | *<* 0*.*001 | 0.33 | [ 0.03,0.64] |  |
| Trained: Vocoded | -0.02 | [-0.03,-0.01] | -3.81 | 1245.00 | *<* 0*.*001 | -0.26 | [-0.39,-0.13] |  |
| Trained: Vocoded + Blurring | -0.04 | [-0.04,-0.03] | -7.43 | 1245.00 | *<* 0*.*001 | -0.50 | [-0.64,-0.37] |  |
| Test: Vocoded | 0.00 | [-0.02,0.01] | -0.51 | 54.31 | 0.612 | -0.07 | [-0.33,0.20] |  |
| Test: Vocoded + Blurring | -0.03 | [-0.06,-0.01] | -3.21 | 48.61 | 0.002 | -0.50 | [-0.81,-0.19] |  |
| Trained: Dutch | -0.02 | [-0.02,-0.01] | -4.94 | 1245.00 | *<* 0*.*001 | -0.22 | [-0.31,-0.13] |  |
| Test: Dutch | -0.03 | [-0.03,-0.02] | -8.89 | 1245.00 | *<* 0*.*001 | -0.40 | [-0.49,-0.31] |  |
| Trained: Vocoded×Test: Vocoded | 0.04 | [ 0.03,0.05] | 6.18 | 1245.00 | *<* 0*.*001 | 0.59 | [ 0.40,0.78] |  |
| Trained: Vocoded + Blurring×Test: Vocoded | 0.04 | [ 0.03,0.05] | 5.99 | 1245.00 | *<* 0*.*001 | 0.57 | [ 0.39,0.76] |  |
| Trained: Vocoded×Test: Vocoded + Blurring | 0.05 | [ 0.04,0.06] | 7.23 | 1245.00 | *<* 0*.*001 | 0.69 | [ 0.50,0.88] |  |
| Trained: Vocoded + Blurring×Test: Vocoded + Blurring | 0.08 | [ 0.07,0.10] | 12.36 | 1245.00 | *<* 0*.*001 | 1.18 | [ 1.00,1.37] |  |
| Trained: Dutch×Test: Dutch | 0.02 | [ 0.01,0.03] | 5.13 | 1245.00 | *<* 0*.*001 | 0.33 | [ 0.20,0.45] |  |
|  |  |  |  |  |  |  |  |  |
| **Random Effects** |  |  |  |  |  |  |  |  |
|  |  |  |  |  |  |  |  |  |
| Within-Group Variance (SD) |  | 0.00 (0.04) |  |  |  |  |  |  |
| Between-Group Variance (SD) |  |  |  |  |  |  |  |  |
|  | Intercept (Participant) | 0.00 (0.06) |  |  |  |  |  |  |
|  | Slope (Vocoded) | 0.00 (0.05) |  |  |  |  |  |  |
|  | Slope (Vocoded + Blurring) | 0.00 (0.06) |  |  |  |  |  |  |
| Correlations |  |  |  |  |  |  |  |  |
|  | Participant × Vocoded | -0.49 |  |  |  |  |  |  |
|  | Participant × Vocoded + Blurring | -0.75 |  |  |  |  |  |  |
| Groups | Vocoded × Vocoded + Blurring | 0.66 |  |  |  |  |  |  |
|  |  |  |  |  |  |  |  |  |
|  | Participant | 38 |  |  |  |  |  |  |
| Observations |  | 1368 |  |  |  |  |  |  |
|  |  |  |  |  |  |  |  |  |
| **Model Metrics** |  |  |  |  |  |  |  |  |
|  |  |  |  |  |  |  |  |  |
| AIC | BIC | R^2^ (Conditional) | R^2^ (Marginal) | ICC | RMSE |  |  |  |
| -4373.39 | -4274.19 | 0.66 | 0.09 | 0.63 | 0.04 |  |  |  |
|  |  |  |  |  |  |  |  |  |

**Table A6**

*Contrast of subject-specific Decoding Accuracy (r) estimated marginal means for Test: Spectral Degradation within levels of Trained: Spectral Degradation. P-values are adjusted using the Bonferroni method.*

| Contrast: Test: Spectral Degradation | | At: Trained: Spectral Degradation | Estimate | 95% CI | SE | df | *t* | *p* |
| --- | --- | --- | --- | --- | --- | --- | --- | --- |
|  |  |  |  |  |  |  |  |  |
| Unprocessed | Vocoded | Unprocessed | 0.00 | [-0.02,0.03] | 0.01 | 54.31 | 0.51 | 1.000 |
| Unprocessed | Vocoded + Blurring | Unprocessed | 0.03 | [ 0.01,0.06] | 0.01 | 48.61 | 3.21 | 0.007 |
| Vocoded | Vocoded + Blurring | Unprocessed | 0.03 | [ 0.01,0.05] | 0.01 | 56.33 | 3.39 | 0.004 |
| Unprocessed | Vocoded | Vocoded | -0.04 | [-0.06,-0.01] | 0.01 | 54.31 | -3.97 | 0.001 |
| Unprocessed | Vocoded + Blurring | Vocoded | -0.01 | [-0.04,0.01] | 0.01 | 48.61 | -1.26 | 0.637 |
| Vocoded | Vocoded + Blurring | Vocoded | 0.02 | [ 0.00,0.05] | 0.01 | 56.33 | 2.59 | 0.036 |
| Unprocessed | Vocoded | Vocoded + Blurring | -0.04 | [-0.06,-0.01] | 0.01 | 54.31 | -3.83 | 0.001 |
| Unprocessed | Vocoded + Blurring | Vocoded + Blurring | -0.05 | [-0.08,-0.02] | 0.01 | 48.61 | -4.45 | *<* 0*.*001 |
| Vocoded | Vocoded + Blurring | Vocoded + Blurring | -0.01 | [-0.03,0.01] | 0.01 | 56.33 | -1.43 | 0.478 |
|  |  |  |  |  |  |  |  |  |

**Table A7**

*Contrast of subject-specific Decoding Accuracy (r) estimated marginal means for Test: Language within levels of Trained: Language. P-values are adjusted using the Bonferroni method.*

| Contrast: Test: Language | | At: Trained: Language | Estimate | 95% CI | SE | df | *t* | *p* |
| --- | --- | --- | --- | --- | --- | --- | --- | --- |
|  |  |  |  |  |  |  |  |  |
| English | Dutch | English | 0.03 | [0.02,0.03] | 0.00 | 1245.00 | 8.89 | *<* 0*.*001 |
| English | Dutch | Dutch | 0.01 | [0.00,0.01] | 0.00 | 1245.00 | 1.63 | 0.104 |
|  |  |  |  |  |  |  |  |  |

**Group Decoding**

**Table A8**

*Model selection for the dependent variable of group Decoding Accuracy (r). Missing values of AIC indicate the model did not converge.*

| Model | df | AIC | Formula | |
| --- | --- | --- | --- | --- |
|  |  |  |  |  |
| 1 | 3 | -4050.93 | 1 | + (1 \| Participant) |
| 2 | 5 | -4035.82 | 1 | + Trained: Spectral Degradation + (1 \| Participant) |
| 3 | 5 | -4053.48 | 1 | + Test: Spectral Degradation + (1 \| Participant) |
| 4 | 7 | -4038.48 | 1 | + Trained: Spectral Degradation + Test: Spectral Degradation + (1 \| Participant) |
| 5 | 11 | -4040.59 | 1 | + Trained: Spectral Degradation × Test: Spectral Degradation + (1 \| Participant) |
| 6 | 16 | — | 1 | + Trained: Spectral Degradation × Test: Spectral Degradation + (Trained: Spectral |
|  |  |  | Degradation \| Participant) | |
| 7 | 16 | -4473.73 | 1 | + Trained: Spectral Degradation × Test: Spectral Degradation + (Test: Spectral |
|  |  |  | Degradation \| Participant) | |
| 8 | 17 | -4462.27 | 1 | + Trained: Spectral Degradation × Test: Spectral Degradation + Trained: Language + |
|  |  |  | (Test: Spectral Degradation \| Participant) | |
| 9 | 17 | -4509.57 | 1 | + Trained: Spectral Degradation × Test: Spectral Degradation + Test: Language + |
|  |  |  | (Test: Spectral Degradation \| Participant) | |
| 10 | 20 | — | 1 | + Trained: Spectral Degradation × Test: Spectral Degradation + Test: Language + |
|  |  |  | (Test: Spectral Degradation \| Participant) + (Test: Language \| Participant) | |
| 11 | 19 | -4495.02 | 1 | + Trained: Spectral Degradation × Test: Spectral Degradation + Trained: Language × |
|  |  |  | Test: Language + (Test: Spectral Degradation \| Participant) | |
| 12 | 43 | -4295.10 | 1 | + Trained: Spectral Degradation × Test: Spectral Degradation × Trained: Language × |
|  |  |  | Test: Language + (Test: Spectral Degradation \| Participant) | |
|  |  |  |  |  |

**Table A9**

*Model formula: Decoding Accuracy R Value* ∼ *1 + Trained: Spectral Degradation* × *Test: Spectral Degradation + Test: Language + (Test: Spectral Degradation | Participant). Standardized parameters were obtained by fitting the model on a standardized version of the dataset. 95% Confidence Intervals (CIs) and p-values were computed using a Wald t-distribution with Kenward-Roger approximation.*

| Parameter | Estimate | 95% CI | *t* | df | *p* | Std. Estimate | Std. Estimate 95% CI |  |
| --- | --- | --- | --- | --- | --- | --- | --- | --- |
|  |  |  |  |  |  |  |  |  |
| (Intercept) | 0.11 | [ 0.10,0.13] | 16.28 | 53.39 | *<* 0*.*001 | 0.22 | [-0.01,0.44] |  |
| Trained: Vocoded | -0.01 | [-0.02,0.00] | -2.26 | 1247.00 | 0.024 | -0.17 | [-0.31,-0.02] |  |
| Trained: Vocoded + Blurring | -0.03 | [-0.04,-0.02] | -5.85 | 1247.00 | *<* 0*.*001 | -0.43 | [-0.57,-0.29] |  |
| Test: Vocoded | 0.00 | [-0.02,0.02] | -0.28 | 50.28 | 0.778 | -0.04 | [-0.36,0.27] |  |
| Test: Vocoded + Blurring | -0.03 | [-0.05,0.00] | -2.46 | 48.08 | 0.018 | -0.42 | [-0.76,-0.08] |  |
| Test: Dutch | -0.02 | [-0.02,-0.01] | -7.01 | 1247.00 | *<* 0*.*001 | -0.24 | [-0.31,-0.17] |  |
| Trained: Vocoded×Test: Vocoded | 0.03 | [ 0.01,0.04] | 3.89 | 1247.00 | *<* 0*.*001 | 0.40 | [ 0.20,0.61] |  |
| Trained: Vocoded + Blurring×Test: Vocoded | 0.03 | [ 0.02,0.05] | 5.04 | 1247.00 | *<* 0*.*001 | 0.52 | [ 0.32,0.73] |  |
| Trained: Vocoded×Test: Vocoded + Blurring | 0.03 | [ 0.02,0.05] | 5.23 | 1247.00 | *<* 0*.*001 | 0.54 | [ 0.34,0.75] |  |
| Trained: Vocoded + Blurring×Test: Vocoded + Blurring | 0.05 | [ 0.04,0.07] | 8.17 | 1247.00 | *<* 0*.*001 | 0.85 | [ 0.64,1.05] |  |
|  |  |  |  |  |  |  |  |  |
| **Random Effects** |  |  |  |  |  |  |  |  |
|  |  |  |  |  |  |  |  |  |
| Within-Group Variance (SD) |  | 0.00 (0.04) |  |  |  |  |  |  |
| Between-Group Variance (SD) |  |  |  |  |  |  |  |  |
|  | Intercept (Participant) | 0.00 (0.04) |  |  |  |  |  |  |
|  | Slope (Vocoded) | 0.00 (0.05) |  |  |  |  |  |  |
|  | Slope (Vocoded + Blurring) | 0.00 (0.06) |  |  |  |  |  |  |
| Correlations |  |  |  |  |  |  |  |  |
|  | Participant × Vocoded | -0.54 |  |  |  |  |  |  |
|  | Participant × Vocoded + Blurring | -0.45 |  |  |  |  |  |  |
| Groups | Vocoded × Vocoded + Blurring | 0.64 |  |  |  |  |  |  |
|  |  |  |  |  |  |  |  |  |
|  | Participant | 38 |  |  |  |  |  |  |
| Observations |  | 1368 |  |  |  |  |  |  |
|  |  |  |  |  |  |  |  |  |
| **Model Metrics** |  |  |  |  |  |  |  |  |
|  |  |  |  |  |  |  |  |  |
| AIC | BIC | R^2^ (Conditional) | R^2^ (Marginal) | ICC | RMSE |  |  |  |
| -4509.57 | -4420.82 | 0.60 | 0.05 | 0.57 | 0.04 |  |  |  |
|  |  |  |  |  |  |  |  |  |

**Table A10**

*Contrast of group Decoding Accuracy (r) estimated marginal means for Test: Spectral Degradation within levels of Trained: Spectral Degradation. P-values are adjusted using the Bonferroni method.*

| Contrast: Test: Spectral Degradation | | At: Trained: Spectral Degradation | Estimate | 95% CI | SE | df | *t* | *p* |
| --- | --- | --- | --- | --- | --- | --- | --- | --- |
|  |  |  |  |  |  |  |  |  |
| Unprocessed | Vocoded | Unprocessed | 0.00 | [-0.02,0.03] | 0.01 | 50.28 | 0.28 | 1.000 |
| Unprocessed | Vocoded + Blurring | Unprocessed | 0.03 | [ 0.00,0.05] | 0.01 | 48.08 | 2.46 | 0.053 |
| Vocoded | Vocoded + Blurring | Unprocessed | 0.02 | [ 0.00,0.05] | 0.01 | 53.41 | 2.57 | 0.039 |
| Unprocessed | Vocoded | Vocoded | -0.02 | [-0.05,0.00] | 0.01 | 50.28 | -2.26 | 0.085 |
| Unprocessed | Vocoded + Blurring | Vocoded | -0.01 | [-0.03,0.02] | 0.01 | 48.08 | -0.72 | 1.000 |
| Vocoded | Vocoded + Blurring | Vocoded | 0.01 | [-0.01,0.04] | 0.01 | 53.41 | 1.61 | 0.337 |
| Unprocessed | Vocoded | Vocoded + Blurring | -0.03 | [-0.05,-0.01] | 0.01 | 50.28 | -3.01 | 0.012 |
| Unprocessed | Vocoded + Blurring | Vocoded + Blurring | -0.03 | [-0.05,0.00] | 0.01 | 48.08 | -2.50 | 0.047 |
| Vocoded | Vocoded + Blurring | Vocoded + Blurring | 0.00 | [-0.02,0.03] | 0.01 | 53.41 | 0.34 | 1.000 |
|  |  |  |  |  |  |  |  |  |
